# Deterioration mechanisms of tuff with surface fractures under freeze–thaw cycles

**DOI:** 10.1038/s41598-024-62886-3

**Published:** 2024-06-11

**Authors:** Runsen Lai, Zizhao Zhang, Jianhua Zhu, Zhiguo Xu, Xiancheng Wei, Xinyu Liu, Binghui Xiong

**Affiliations:** 1 College of Geology and Mining Engineering, Xinjiang University, Urumqi, 830017 China; 2 Research Base of Xinjiang University, National Key Laboratory of Intelligent Construction and Healthy Operation and Maintenance of Deep-Earth Engineering, Xinjiang University, Urumqi, 830017 Xinjiang China; 3 Xinjiang Geological Environment Monitoring Institute, Urumqi, 830099 Xinjiang China; 4 Xinjiang Uygur Autonomous Region Geological and Mineral Exploration and Development Bureau, Fourth Geological Brigade, Altay, 836500 Xinjiang China

**Keywords:** Freeze–thaw cycles, Surface fractured rock mass, Prefabricated joint, Mechanical property, Fractures morphology, Natural hazards, Engineering

## Abstract

In practical engineering, the development of surface cracks is one of the most important reasons for the destruction of the rock mass, and the development of complex morphology fractures on the rock mass surface significantly influences rock mass mechanics. This paper addresses the freeze–thaw damage issue in rock mass containing surface fractures in cold regions. Tuffs and a control group are selected as research samples, with the control group being prefabricated surface jointed specimens with an inclination angle of 70° and a fracture depth ratio d (crack depth) /t (sample width) of 0.26. The study analyzes the mass, wave velocity loss, macro-microcosmic fracture damage morphology, and mechanical properties of the two specimen groups through laboratory freeze–thaw cycle tests, uniaxial compression tests, and scanning electron microscopy examinations. The results show an overall decrease in mass, wave velocity, and uniaxial compressive strength as the cycle number increases, with the prefabricated jointed group samples showing more significant changes. However, the two specimen groups exhibit different macroscopic failure fracture states. In addition, scanning electron microscopy images illustrate that after freeze–thaw cycles, the large rock mass particles break into smaller fragments, resulting in looser particle arrangements and a transition from initial surface cementation to point contact., which weakens the compressive strength of the rock mass. The paper also explains the mechanism of the diminishing impact of freeze–thaw cycles on the strength of the rock mass after a certain number of cycles. The research outcomes hold significant reference value for engineering construction in cold regions.

## Introduction

The damage of rock mass is often associated with joints, fractures, or other structural surfaces. Natural rock mass inherently contains numerous joints, and these masses experience cyclic loading from frost heaving force under the influence of freeze-thaw cycles. This action causes subsequent expansion and closure of joints and fractures, resulting in the coherent structural surfaces, which will lead to the destruction and destabilization of the rock mass. Studies on rock mass destruction and destabilization primarily show that rock mass damage under freeze-thaw cycles is mainly attributed to cyclic frost heaving force. Wettlaufer et al.^1^ indicate an interface effect within the rock mass. During the freezing process, a portion of unfrozen water located at joints, pores, or fractures migrates to the bottom due to the frozen water, thereby continually increasing water pressure and leading to downward fracture expansion. Setzer^2^ discovers that the water-ice phase transition induces a volume expansion of about 9%, with some water in the pores and fractures remaining liquid after freezing. Liu et al.^3^ propose that frost heaving force results from the common phase transition of in-situ water in rock mass fractures and migrating water, causing fractures to expand and ultimately leading to rock mass damage. The above studies illustrate the failure mechanism of rock mass under freeze-thaw cycles, a critical consideration in rock mass engineering where safety and durability requirements are increasingly stringent. Rock mass and soil experience year-round low temperatures and freeze-thaw cycles in alpine regions; thus, investigating mechanical properties under extreme environmental conditions becomes highly significant.

In practical engineering, rock masses exhibit a higher occurrence of joints, fractures, and various structural surfaces following the construction of tunnels, rocky slopes, etc. The intricate nature of jointed and fractured rock masses significantly influences the safety and stability of rock mass engineering. The focus of research on fractured rock masses primarily includes three categories: natural joints and fractures within the protolith, prefabricated joints and fractures within the protolith, and prefabricated joints and fissures within similar materials. Rodríguez et al.^4^ conduct loading and unloading splitting tests on marble with microfractures, revealing fracture extension evolution in different conditions through the acoustic emission (AE) method. Bao et al.^5^ uncover the anisotropic freeze-thaw damage law in rock masses by producing protolithic jointed and fractured rock masses with different bedding angles and conducting freeze-thaw and uniaxial tests. Pan et al.^6^ investigate the uniaxial compressive mechanical properties of marble with prefabricated fractures containing various fillings. Li et al.^7^ explore the acoustic emission law and seismic source mechanism of granite specimens with prefabricated fractures during hydraulic fracturing, noting shock source concentration at the tips of prefabricated cracks. Wang et al.^8^ conduct true triaxial loading and unloading tests on similar materials of rock mass specimens with prefabricated joints and fractures, identifying deformation and damage characteristics. Zhu^9,10^ determines the change law of the stress intensity factor under compressive loading tests on similar materials of rock mass specimens with prefabricated joints and fractures, which include single and double cracks.

Synthesizing the results of the above research, it is evident that cyclic freeze-thaw force induces gradual deterioration and destabilization of rock masses. The initiation of rock mass body damage often emerges from surface fractures, posing challenges to engineering construction in cold regions. It becomes particularly urgent to delve into the laws governing the degradation and damage of rock masses containing surface fractures under the influence of freeze-thaw cycles. Previous studies show that the test rock mass typically features penetrating prefabricated fractures, assuming fractures that penetrate the entire rock mass. Nonetheless, actual engineering scenarios commonly involve non-penetrating fractures (surface fractures)^11^, where fracture expansion occurs within the rock mass in three-dimensional space. The suppose of penetrating prefabricated fractures neglects the depth dimension of the fracture, potentially missing crucial three-dimensional information about the rupture^12^. R.H.C. Wong et al.^13,14^ establish a close relationship between the expansion mode of surface fractures and the fracture depth ratio (d/t) through the examination of rock-like materials. Nakamura^15^ measures the surface temperature of tuff fractures, establishing the relationship between frost heaving force and fracture temperature. Huang et al.^16^ demonstrate that the penetration of three-dimensional surface cracks is more intricate than two-dimensional penetration cracks using PMMA transparent materials. Hence, there is a need to shift the research focus to surface fractures to gain comprehensive insights into the laws governing rock mass fracture extension.

Presently, the researchs about the properties of rock mass with prefabricated joints under freeze-thaw action predominantly center on the mechanical strength of the rock mass^16–21,23,24^, followed by alterations in the microstructure^22–27^. Other investigations encompass the angular influence of prefabricated joints^17,18^, penetration degree^17^, and the comparison of different lithologies^20^ etc. The associated research methods primarily revolve around laboratory tests, including uniaxial compression tests^16–19^, triaxial tests^20,21,23^, acoustic emission tests^19^, polarized light microscopy^22^, scanning electron microscopy^23,24^, nuclear magnetic resonance (NMR), CT scan, etc.^25^, and CT scanning^26,27^, etc. This paper will employ freeze-thaw cycle tests, uniaxial compression tests, and SEM tests to investigate prefabricated surface jointed rock mass samples.

Limited studies have delved into the cracking process and expansion laws of surface fractured rock masses, particularly the correlation between macroscopic mechanical strength characteristics and deformation characteristics of surface fractured rock masses under the extreme conditions of freeze-thaw cycles. The actual projects often involve more joints and fractures on the rock mass surface, significantly influencing the rock's deterioration due to freeze-thaw cycles. This paper fabricates surface jointed specimens and subjects them to uniaxial compression tests after different freeze-thaw cycles. Along with research on macroscopic crack extension and microscopic analysis using scanning electron microscopy, this study aims to unravel the freeze-thaw damage laws of tuff and investigate the mechanical properties and crack extension of fractured rock mass under freeze-thaw cycle conditions.

## Laboratory tests

This research studies the rocks extracted from the Camel's Hump on the western side of Altay City's downtown area in Xinjiang. The region experiences an average annual temperature of 4.80 °C, with the highest temperatures occurring in July (averaging around 20 °C) and the lowest temperatures in January (with an average of – 30 ℃ and an extreme minimum of − 41.7 °C). Winter spans a considerable duration of 127–169 days, creating a significant temperature differential between seasons. Given these conditions, the rocks in this area are highly influenced by the freeze-thaw action.

### Specimen preparation

The test tuff is characterized mainly by tuff lithology and obtained from the collapsed slope of Camel's Hump Scenic Area in Altay City, Xinjiang. Specimens are prepared into Ф50 × 100 mm rock samples. Utilizing a rock wave velocity detector, samples are screened to ensure an average wave velocity of 4.448 m/s and a rock density of 2786 kg/m^3^, aligning with the grouping criteria for the tests.Based on the actual survey, the time when the rock is most strongly affected by freezing and thawing is mainly concentrated in the alternation of winter and spring, and the freezing and thawing period is about 1 month.

**Figure 1:**
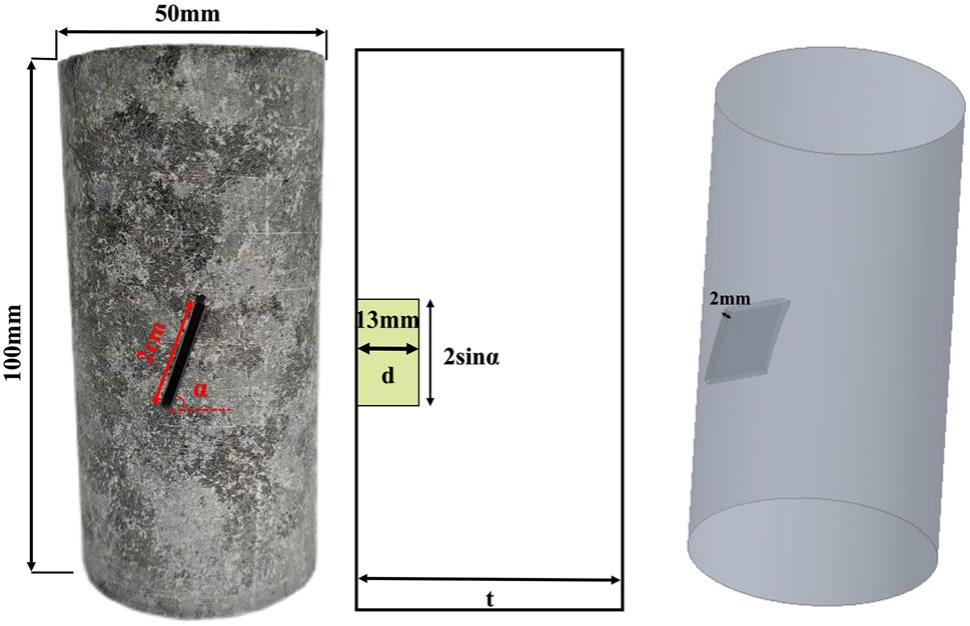
Schematic diagram of tuff specimen dimensions.

To prevent damage to the rock mass structure around the joints during the waterjet machining process, an automated machine tool is employed to create non-through joints. These joints, with a thickness of approximately 1.5 mm, length of 20 mm, and a depth (d) of 13 mm (see Fig. 1), are statistically determined based on the most prevalent structural plane at an inclination angle of 37°∠70°, identified through joint statistics of the collapsed hazardous rock mass^28^. The chosen inclination angle for the prefabricated joints is 70°. Studies indicate that when the crack depth ratio (d/t) exceeds 1/3^13,14,29^, the fracture penetrates the specimen in the thickness direction, creating a stress state close to the two-dimensional state. To simulate the expansion process of surface fractures in a three-dimensional stress state, this study chooses a crack depth ratio of d/t=0.26. This ensures that fracture expansion occurs entirely within the specimen, maintaining a three-dimensional stress state. Following specimen processing, those of superior mass are selected for testing.

### Test scheme

#### Freeze-thaw cycle test

**Figure 2:**
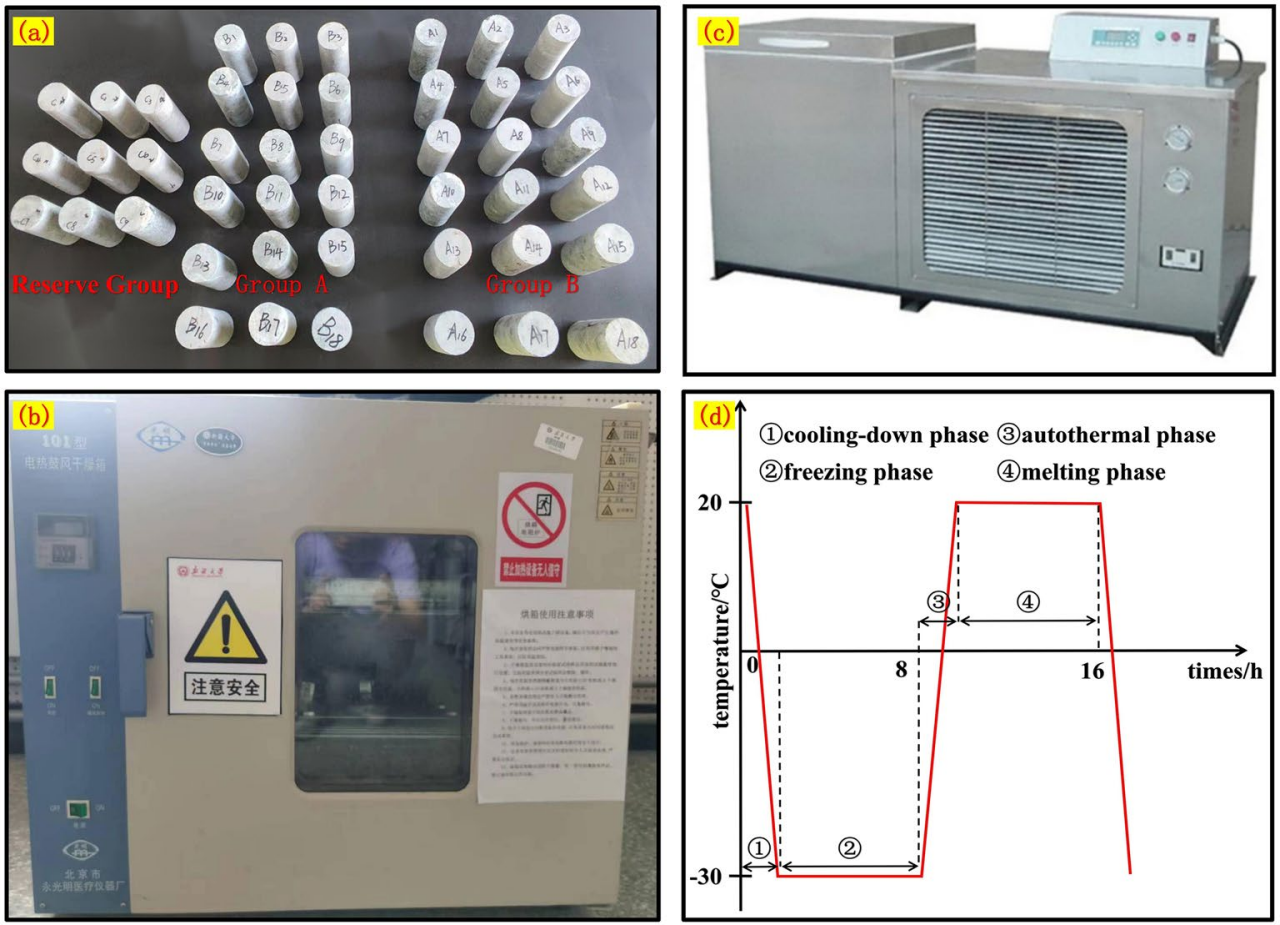
Freeze–thaw cycle test process.

The test specimens are categorized into two groups: original rock samples (Group A) and prefabricated jointed rock samples (Group B) (Fig. 2a). Each group is further subdivided into seven sets, each comprising three specimens, resulting in a total of 42 standard specimens for testing. Freeze-thaw cycles are conducted 0, 1, 3, 5, 10, 20, and 30 times, with the freeze-thaw temperature set to – 30 °C~20 °C to emulate the climatic conditions of the site. Both Group A and Group B specimens are initially placed in an oven at 105 °C for 24 h (Fig. 2b). Following the recording of dry specimen weights, they are subjected to a freeze-thaw cycler (Fig. 2c), maintaining a temperature of (20 °C ± 1 °C) for 8 hours, followed by stabilization at (−30 °C ± 1 °C) for 8 hours, constituting a 16-hour cycling cycle (Fig. 2d). At the conclusion of each freeze-thaw cycle set, the specimens are retrieved and placed in a drying box. Various parameters such as mass and wave speed are measured, and macroscopic observations of freeze-thaw damage to the specimen's appearance are also recorded.

#### Uniaxial compression test

**Figure 3:**
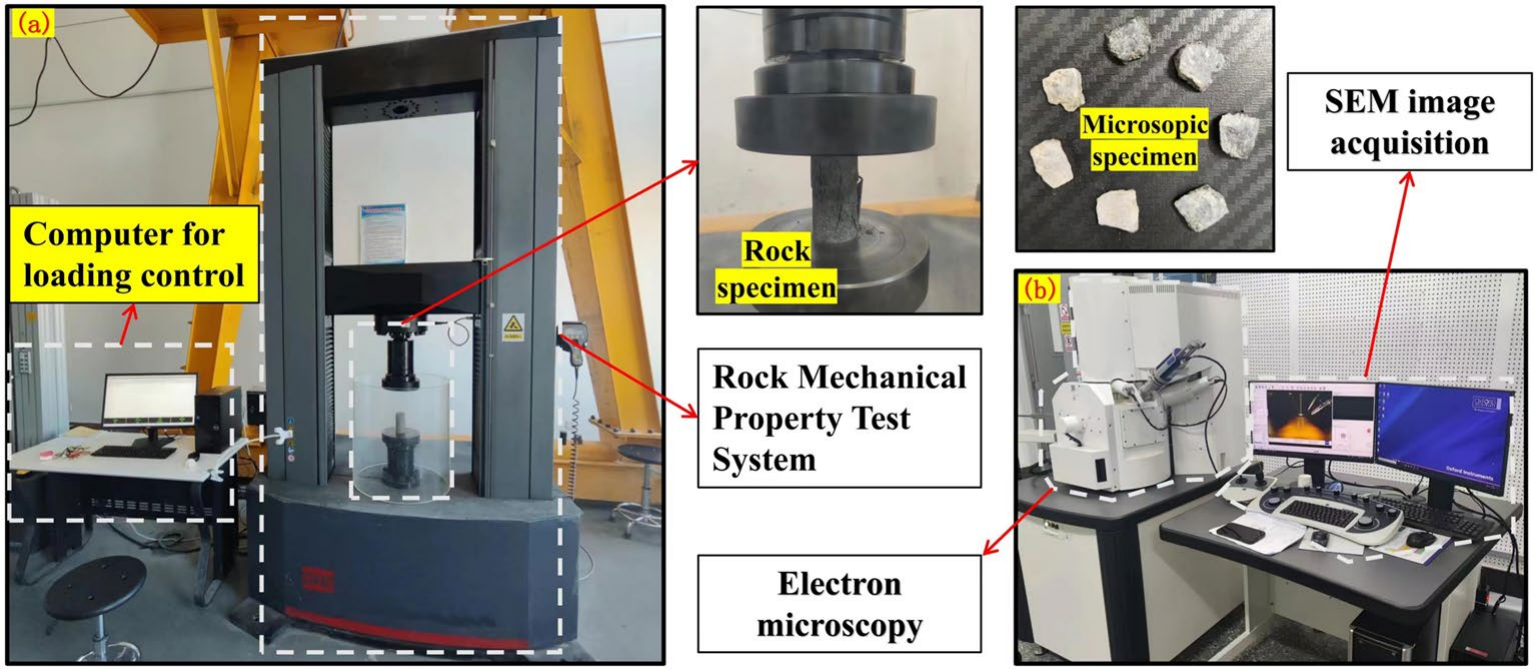
Rock specimen test system. (**a**) Mining rock mechanical property test system. (**b**) Scanning electron microscopy.

After each freeze-thaw cycle set and 24-hour drying, three corresponding specimens are subjected to uniaxial compression tests. Using the MTS E45.605 mining rock mechanical property test system (Fig. 3a), the rock samples undergo uniaxial compression tests at a loading rate of 0.1 MPa/s until specimen destruction. This analysis aims to explore the correlation between macroscopic mechanical properties, surface fractures, and the number of freeze-thaw cycles. Abnormal data are excluded, and the remaining results are averaged for each group.

#### Scanning electron microscopy test

Specimens that completed the freeze-thaw cycle test and uniaxial compression test undergo testing and analysis through the scanning electron microscopy (SEM) method to observe damage in typical rock samples (see Fig. 3b).

## Tests results and analysis

Based on the outlined test program, both the original rock samples in Group A and the prefabricated jointed rock samples in Group B undergo freeze-thaw cycles and subsequent mechanical tests. The corresponding results and analyses are detailed below.

### Changes in specimen mass after freeze–thaw cycles

Post freeze-thaw cycles, alterations in specimen mass, which is a crucial indicator of freeze-thaw damage, are observed. The initial mass of the dried specimen (m_0_) is weighed after grouping, and the dry mass before and after each cycle is compared to derive the rate of mass change (R_x_), characterizing the specimen's mass variation.

The equation for the rate of change in mass for different cycle numbers is given by:

1 $$ R_{mn} = \frac{{m_{0} - m_{n} }}{{m_{0} }} \times 100\% $$

where, R_mn_ represents the rate of change in mass for different cycle numbers, m_0_ represents the initial rock sample mass in g and m_n_ represents the rock sample mass in g corresponding to n times cycle.

**Figure 4:**
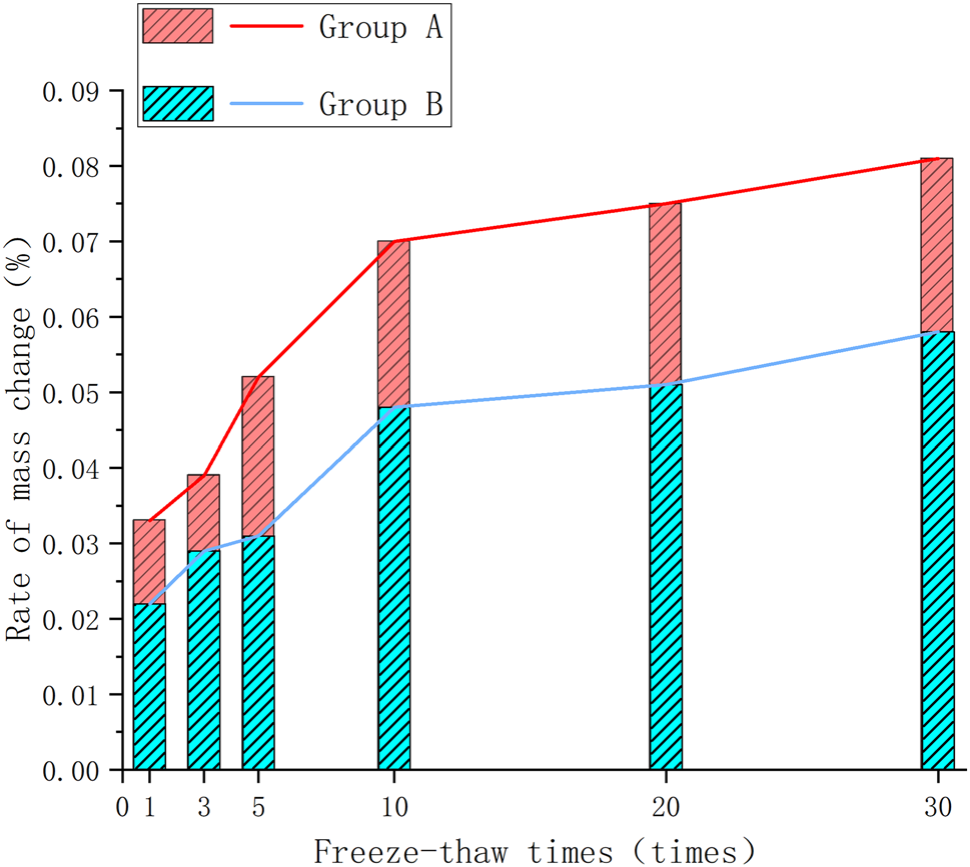
Changes in mass of group A and B specimens after freeze–thaw cycles.

As indicated in Table 1, the mass of both specimen groups gradually decreases with an increase in the number of cycles, albeit with a relatively small overall change. Calculate the freeze-thaw mass rate of change and plot the graph (Fig. 4). The results demonstrate a gradual increase in the mass change rate along with the increasing numbers of freeze-thaw cycles for the two sample groups. The magnitude of mass loss exhibits a trend of initial increase followed by a subsequent decrease.

**Table 1:** Average mass change under freeze–thaw cycles.

Group A of original rock samples					Group B of prefabricated jointed rock samples				
Cycling numbers	Pre-freeze–thaw cycles (g)	Post-freeze–thaw cycles (g)	Mass difference (g)	Mass change rate (R_x_)	Cycling numbers	Pre-freeze–thaw cycles (g)	Post-freeze–thaw cycles (g)	Mass difference (g)	Mass change rate (R_x_)
1	580.35	580.23	0.13	0.022%	1	528.32	528.15	0.17	0.033%
3	563.85	563.69	0.16	0.029%	3	573.94	573.72	0.22	0.039%
5	555.27	555.09	0.17	0.031%	5	508.30	508.04	0.26	0.052%
10	513.97	513.73	0.25	0.048%	10	535.96	535.59	0.38	0.070%
20	573.90	573.61	0.29	0.051%	20	552.36	551.95	0.41	0.075%
30	539.48	539.16	0.31	0.058%	30	536.73	536.30	0.44	0.081%

Notably, at the 10th cycle, with a faster increase before and a slower increase afterwards. Post 10 cycles, mass loss exhibits a gradual increase, and mass change rate levels off in the later cycles. This suggests that initial test cycles have a less apparent effect on the dense specimen, with subsequent cycles inducing fatigue damage, particle shedding, and pore fracture expansion. At the 10th cycle, evident mass decline occurs, marking the start of surface particle shedding. Given that the tuff specimen is relatively dense, the freeze-thaw effects on the specimen surface are limited. Therefore, the mass change rate tends to stabilize after the 10th cycle.

Comparative analysis between Groups A and B shows a significantly smaller mass change rate in Group A. Moreover, at the 5th cycle, Group B exhibits an apparent sudden increase in mass change rate, indicating that prefabricated surface joints have caused damage to the rock mass structure. The freeze-thaw impact became more pronounced, causing not only surface particle detachment but also shedding within and around the prefabricated surface joint fractures.

### Changes in specimen wave velocity after freeze–thaw cycles

As per the previous discussion, the equation defining the change rate of wave velocity for different cycle numbers is expressed as follows:

2 $$ V_{{{\text{vn}}}} = \frac{{P_{0} - P_{n} }}{{P_{0} }} \times {\text{100\% }} $$

where, V_vx_ represents the change rate of wave velocity for different cycle numbers, P_0_ represents the initial wave velocity of the rock sample in km/s, and P_n_ represents the wave velocity of the rock sample in km/s corresponding to n times cycle.(The wave speed here is the longitudinal wave speed.)

**Figure 5:**
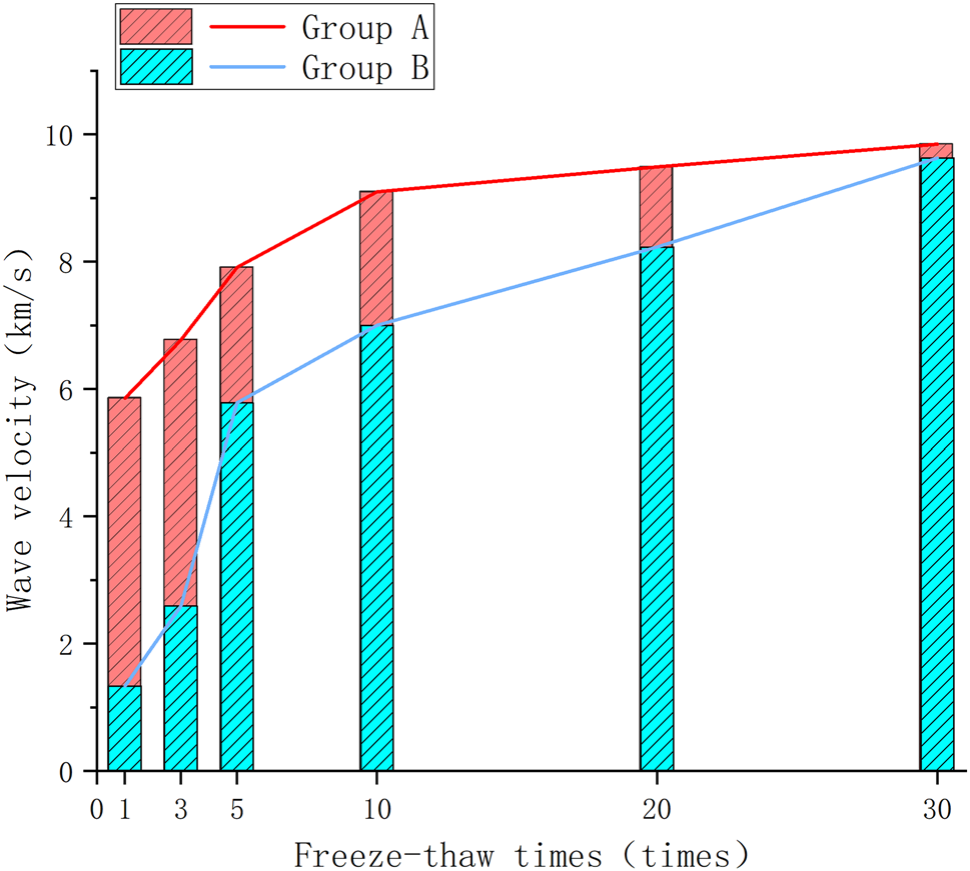
Changes in wave velocity of group A and B specimens after freeze–thaw cycles.

Consistent with the law of mass change, the wave velocity of both sample groups gradually decreases (refer to Table 2). As illustrated in Fig. 5, with an increase in the number of cycles, the rate of change in wave velocity for both groups exhibits a gradual increase, rate of change of wave velocity increases levelling off. As the freeze-thaw cycles accumulate, the change rate in wave velocity tends to gently ascend. During freeze-thaw cycles, the rock's internal pores are compressed by frost heaving forces, leading to pore expansion, a decrease in internal densification, and consequently, a gradual reduction in wave velocity. The change rate in wave velocity increases progressively during this process.

**Table 2:** Average wave velocity change under freeze–thaw cycles.

Group A of original rock samples					Group B of prefabricated jointed rock samples				
Cycling numbers	Pre-freeze–thaw cycles (km/s)	Post-freeze–thaw cycles (km/s)	Wave velocity difference (km/s)	Change rate of wave velocity (V_x_)	Cycling numbers	Pre-freeze–thaw cycles (km/s)	Post-freeze–thaw cycles (km/s)	Wave velocity difference (km/s)	Change rate of wave velocity (V_x_)
1	4.688	4.626	0.06	1.33%	1	3.704	3.487	0.22	5.85%
3	4.479	4.363	0.12	2.59%	3	4.027	3.755	0.27	6.77%
5	4.226	3.981	0.24	5.78%	5	4.288	3.949	0.34	7.91%
10	4.167	3.875	0.29	7.00%	10	3.776	3.432	0.34	9.10%
20	4.801	4.405	0.40	8.23%	20	4.511	4.083	0.43	9.49%
30	4.029	3.641	0.39	9.63%	30	4.085	3.683	0.40	9.85%

Observing the wave velocity changes in groups A and B, it is evident that the change rate in wave velocity in group A is smaller than in group B. However, the change in wave velocity in group B changes more smoothly. In group A, at the 5th freeze-thaw cycle, which increased rapidly before and then slowly thereafter. This surge is attributed to the initial balance in pore expansion in the original rock samples during the first three cycles, and when the cycles reach five, this balance is disrupted, leading to substantial pore expansion and a consequent decrease in rock mass densification. In contrast, the prefabricated surface joints in group B have disturbed the balanced pore state within the rock mass body. Consequently, the pore spaces in group B are more susceptible to expansion and extension, resulting in a smoother change in wave velocity compared to the more abrupt changes observed in group A.

### Apparent damage characteristics after freeze–thaw cycles

**Figure 6:**
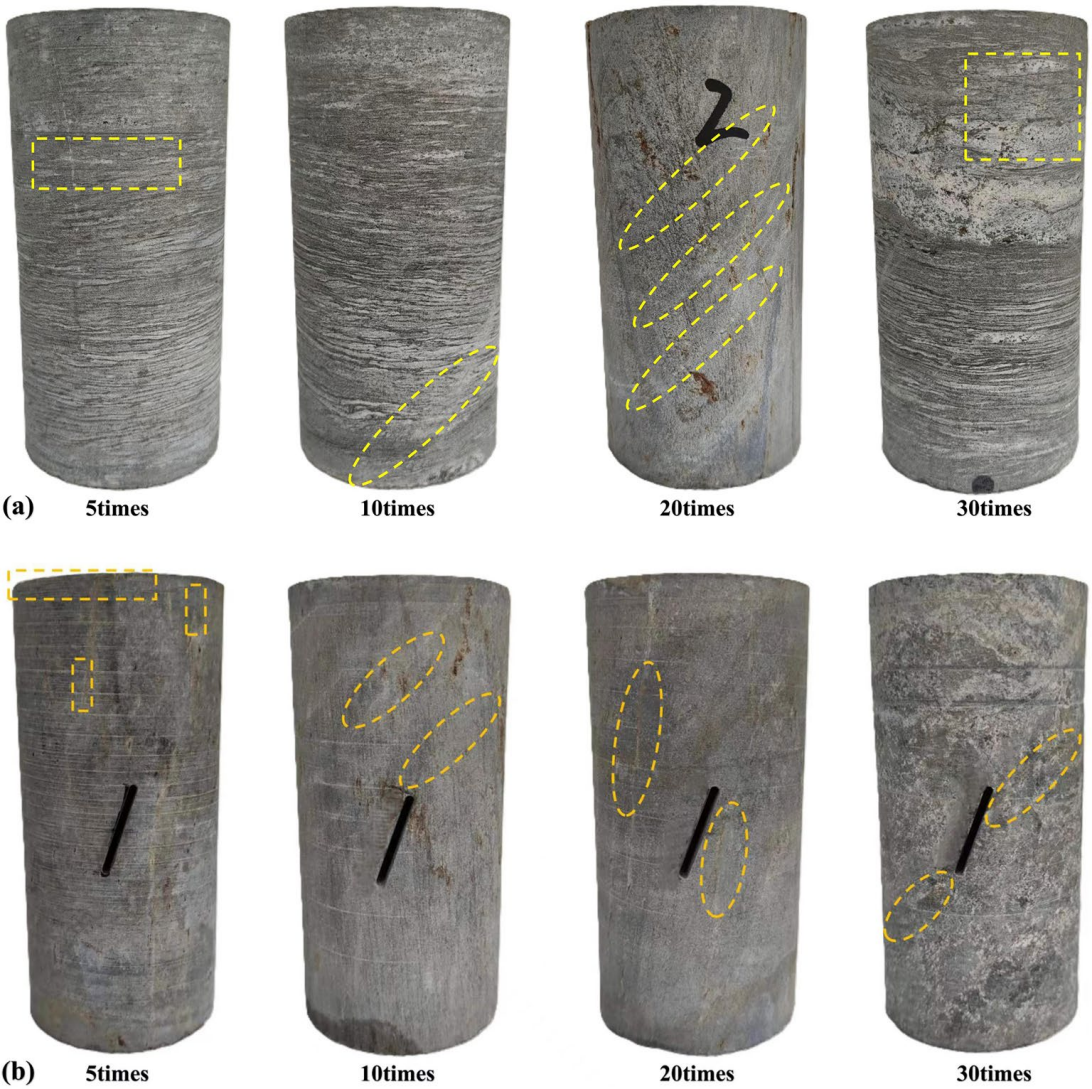
Apparent characteristics of Group A and B specimens not subjected to freeze–thaw cycles.

According to the freeze-thaw cycle test process, both Groups A and B exhibit noticeable surface changes after five cycles, with no chunk falling out. Therefore, typical samples with freeze-thaw cycles of 5, 10, 20 and 30 times were selected for comparative analysis and research. Figure 6 is a sample without freeze-thaw cycle.

**Figure 7:**
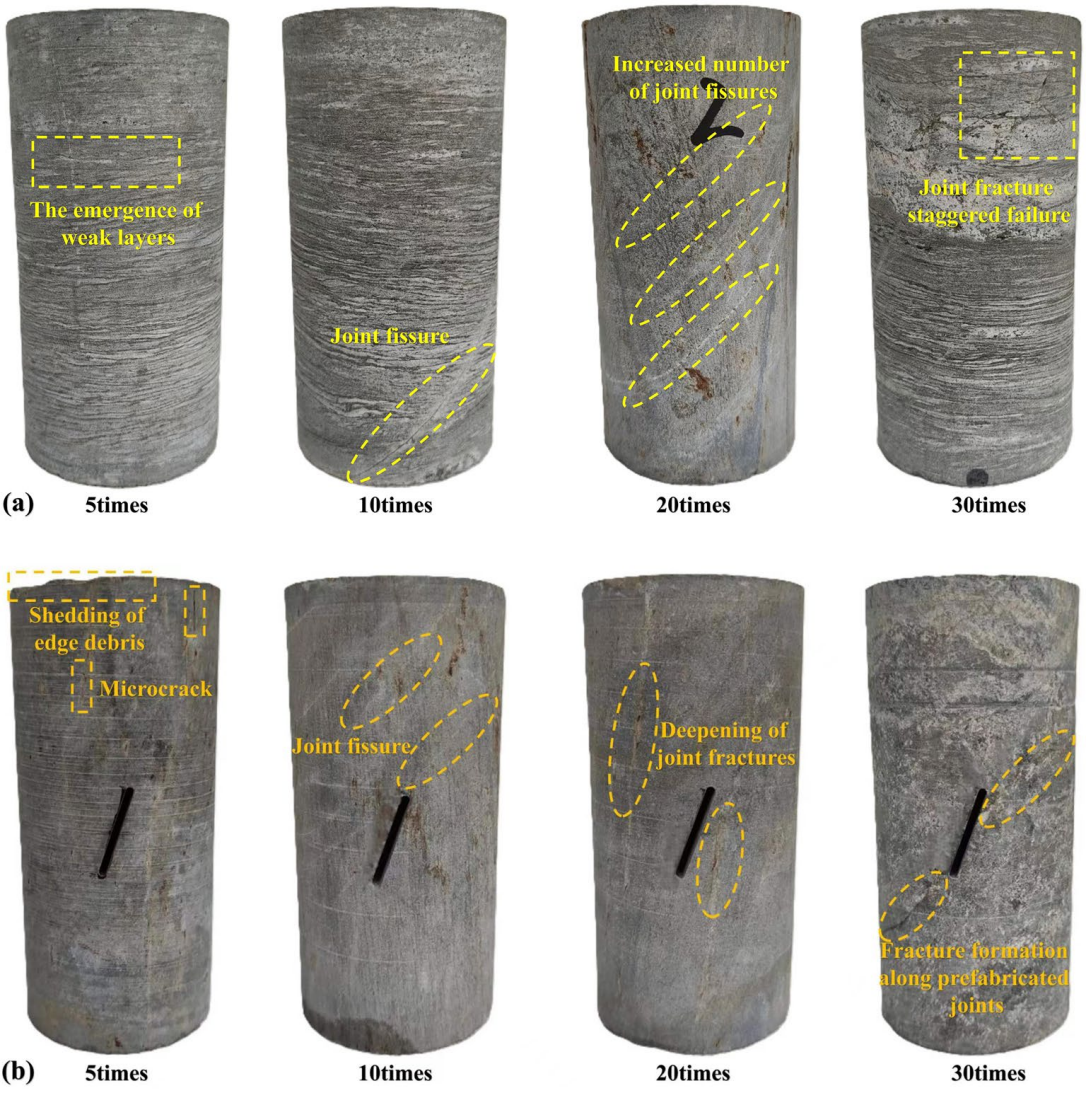
Apparent damage characteristics of group A and B after freeze–thaw cycles.

As shown in Fig. 7a, with the increase of freeze-thaw cycle number, the joint cracks on the surface of Group A specimens increase, gradually intertwining and forming damage on the surface. When the cycle number reaches five times, the weak layers in the direction of bedding on the specimen surface begin to emerge, becoming rougher compared to the original specimen. Subsequently, at ten cycles, evident joint fractures emerge, and by the twentieth cycle, these fractures increase, intensifying the freeze-thaw-induced damage. At thirty cycles, these fractures intersect and develop into approximately 0.4 mm wide cracks, indicative of advanced damage. Since Group B consists of prefabricated jointed rock specimens, the freeze-thaw effects are more pronounced than in Group A. In Fig. 7b, minimal debris detachment and small cracks are visible at the edge of the specimens after five cycles. By ten cycles, several groups of joint cracks form, deepening by the twentieth cycle. At thirty cycles, fractures develop at both ends of the specimen along the direction of the prefabricated joints. However, due to the dense nature of tough specimens, the specimen does not show overall damage.

### Analysis of mechanical properties of rock mass

**Figure 8:**
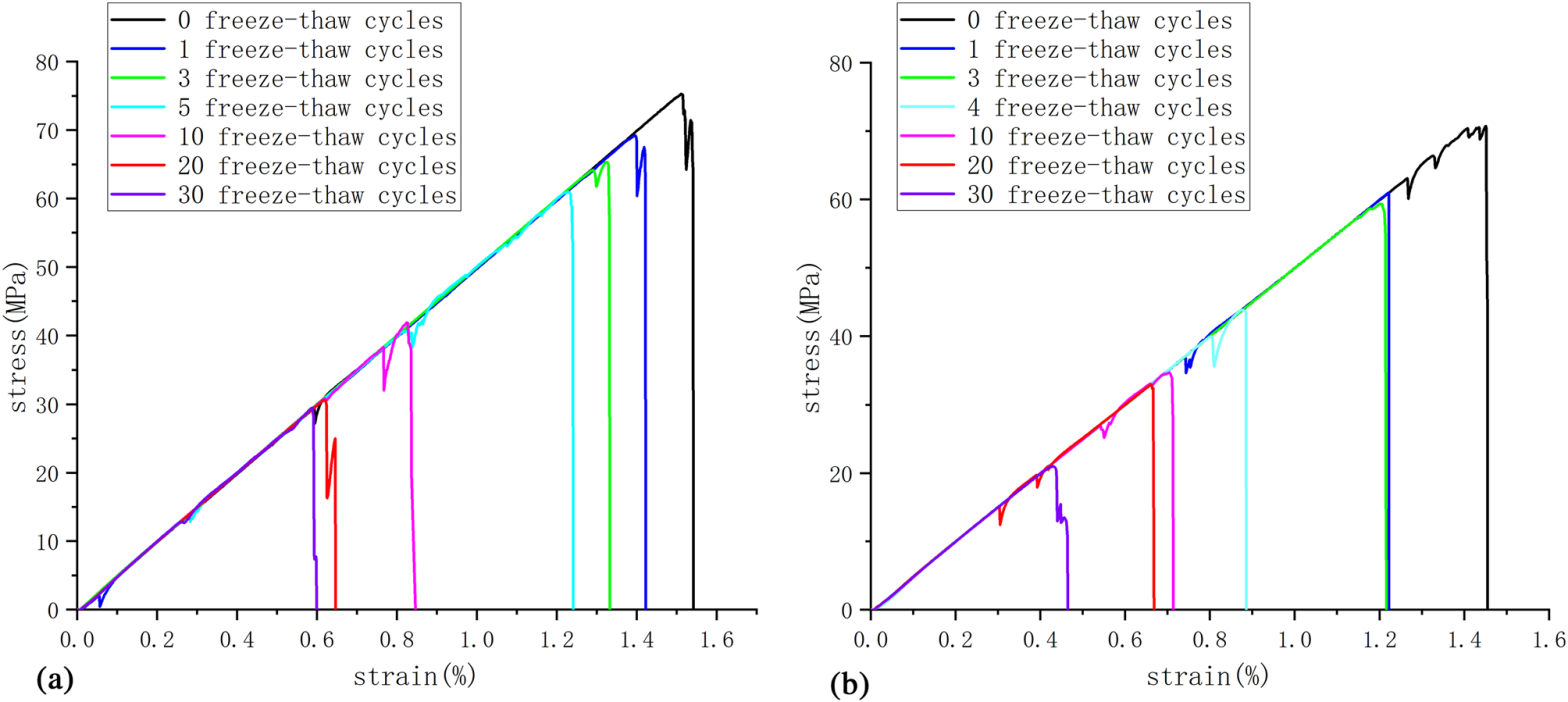
Stress–strain curves under different numbers of freeze–thaw cycles. (**a**) Group A. (**b**) Group B.

Moving to the characterization of uniaxial compressive strength, tests using the MTS E45.605 mining rock mass mechanical property test system are conducted on both Groups A and B for 0, 1, 3, 5, 10, 20, and 30 freeze-thaw cycles. The loading rate was 0.1 MPa/s until specimen destruction, with the test system automatically collecting and storing data. The stress-strain curves obtained (Fig. 8) reveal four stages in the uniaxial compression process: compaction, elastic deformation, plastic deformation, and destruction.

The peak strength of uniaxial compression decreases with increasing freeze-thaw cycles the expansion of microfractures and the increase of soft structures within the rock mass. The compaction phase on the stress-strain curves of both sets of specimens is extended after being subjected to freeze-thaw cycles, indicating that the freeze-thaw cycles create more micropores. The elastic phase is approximately a straight line, and there are more fluctuations during the plastic deformation phase, which is attributed to the more scattered distribution of joints and pores within the rock mass. Curves exhibit vertical trends in the destructive phase, signifying brittleness after freeze-thaw cycles.

Comparing stress-strain curves for Groups A and B, it became evident that prefabricated jointed specimens in Group B are notably affected by freeze-thaw action. Substantial decreases in uniaxial compressive strength are observed in Group A after ten freeze-thaw cycles, while Group B experience this decline after only five cycles. This suggests that prefabricated joint specimens in Group B generate more microporosity in the rock mass after reaching initiation stress. Pores within rocks intersect and penetrate, resulting in damage and peak strength. As the number of freeze-thaw cycles increases, the stress is accompanied by a more minor strain.

### Analysis of uniaxial compressive strength characteristics of rock mass

**Figure 9:**
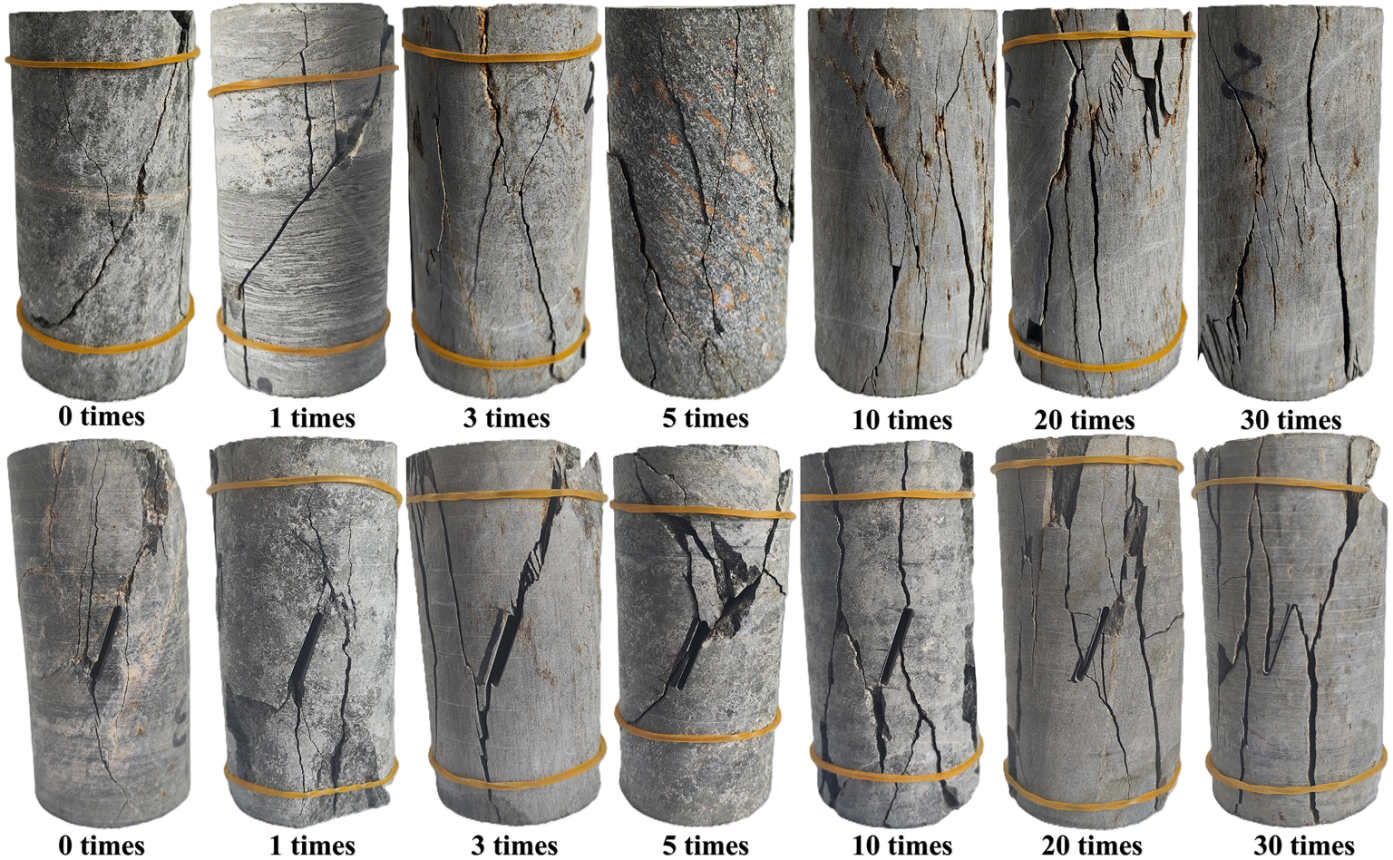
Rock mass fractures damage morphology under different numbers of freeze–thaw cycles.

The rock mass rupture is the macroscopic manifestation of the cumulative deformation and destruction of microfabrication during the process of stressing, resulting from the sprouting and expansion of microcracks until their connectivity is achieved^30^. Due to the difficulty in accurately recording the fracture process of freeze-thawed rock mass cracks during uniaxial compression tests, representative damage morphologies under loading, as shown in Fig. 9, are selected for analysis.

**Figure 10:**
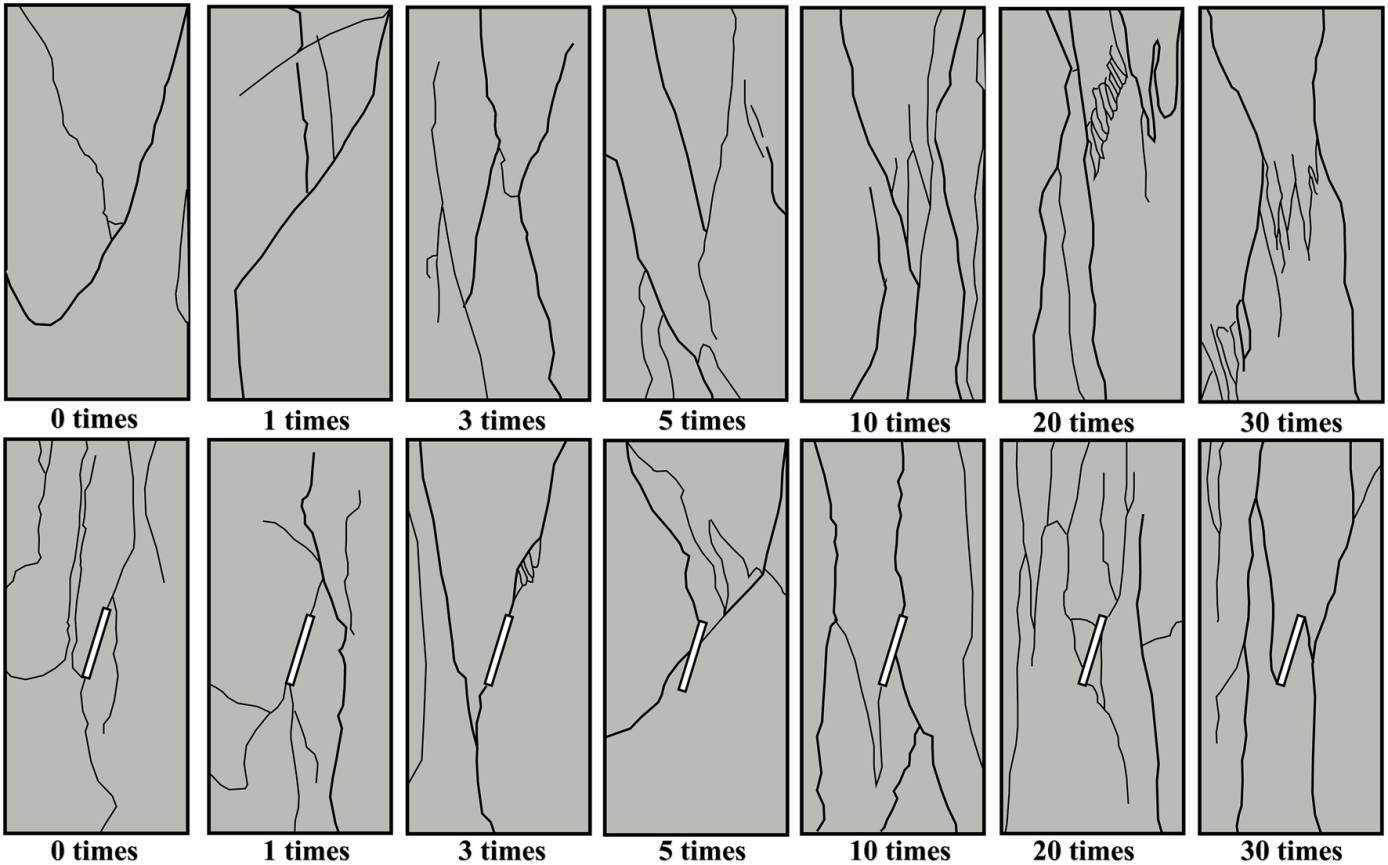
Sketches of rock mass fractures damage morphology.

Sketches of rock mass fractures are created to enhance the observation of the broken ring pattern of rock mass fractures (Fig. 10). With an increasing number of freeze-thaw cycles, the fractures on the specimen's surface under uniaxial compression gradually multiply. The fractures on the original specimen's surface are predominantly penetrating cracks, surrounded by numerous secondary fractures mostly oriented vertically in the loading direction. At 20 and 30 freeze-thaw cycles, secondary fractures increase, showing a different extension pattern compared to specimens without freeze-thaw cycles. The influence of freeze-thaw cycles leads to the appearance and aggregation of joints and fractures inside the rock mass, resulting breakage and the formation of multi-group wing fractures.

In contrast, energy release in prefabricated joints reduces the number of secondary fractures around the main fractures. However, the number of cracks increases similarly with the freeze-thaw cycle. Prefabricated jointed specimens show obvious shear damage at the prefabricated joint face under uniaxial compression, resulting in slip damage along the precast joint face and the formation of wing-shaped fractures at the joint's end. However, at 20 and 30 freeze-thaw cycles, fractures display anti-wing-shaped patterns, similar to those observed by Wong, R. H. C. et al.

**Figure 11:**
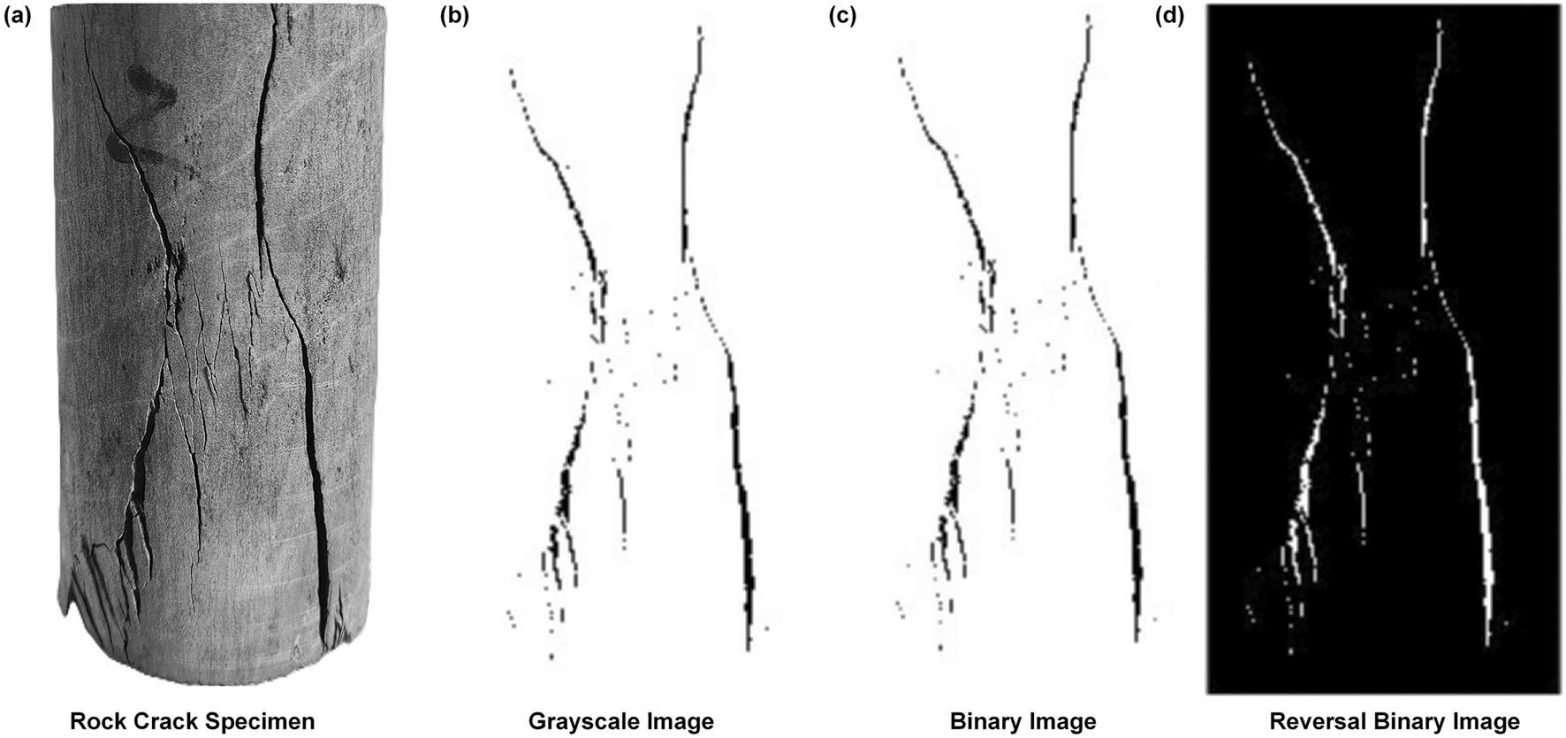
Process diagram of 2D digital image processing of rock mass cracks.

The two-dimensional digital image processing of the rock mass crack image in Fig. 9 is carried out, and the processed image is transferred into the Matlab software plug-in Fraclab to calculate the fractal dimension. Figure 11 is a two-dimensional digital image processing process.

**Figure 12:**
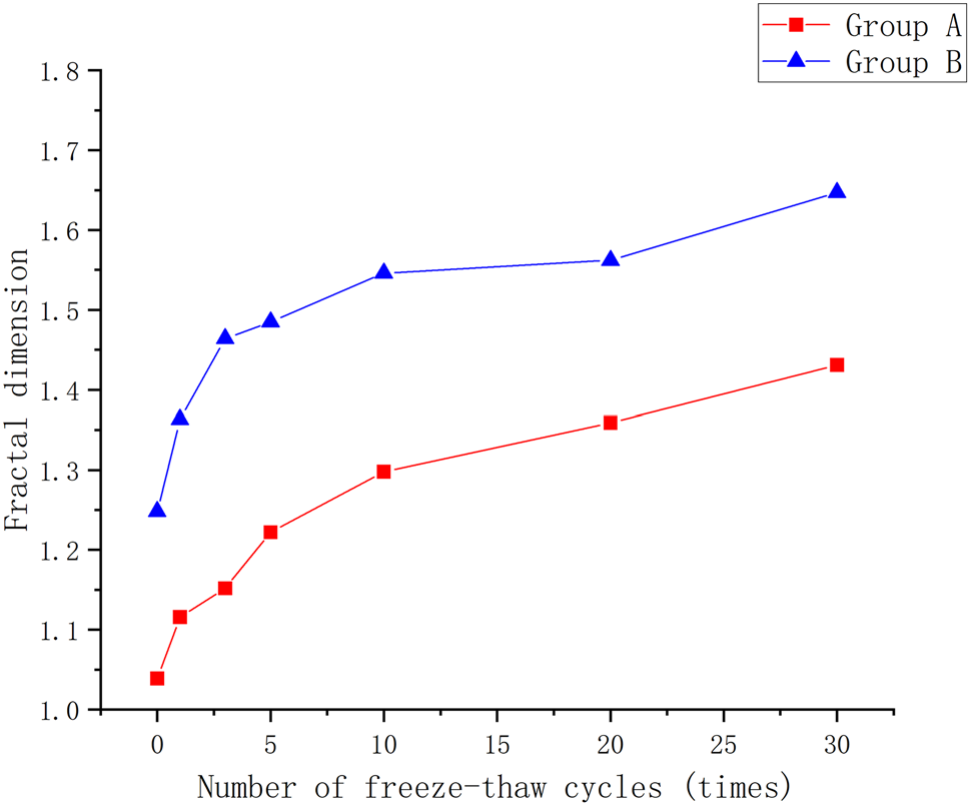
Variation of fractal dimension with different number of freeze–thaw cycles.

The fractal dimension is used to verify the change rule of cracks in rock specimens with the increase of the number of freeze-thaw cycles, and the increase of fractal dimension represents the increase of the complexity within the image, which is calculated to obtain the change map of the fractal dimension of the rock specimens under different numbers of freeze-thaw cycles (Fig. 12). It can be seen that as the number of freeze-thaw cycles increases, the fractal dimension of the sample gradually increases, and the overall fractal dimension of the prefabricated joint sample group (group B) is larger than that of the original sample (group A), which is consistent with the above crack change law.

### Scanning *electron* microscopy test results

**Figure 13:**
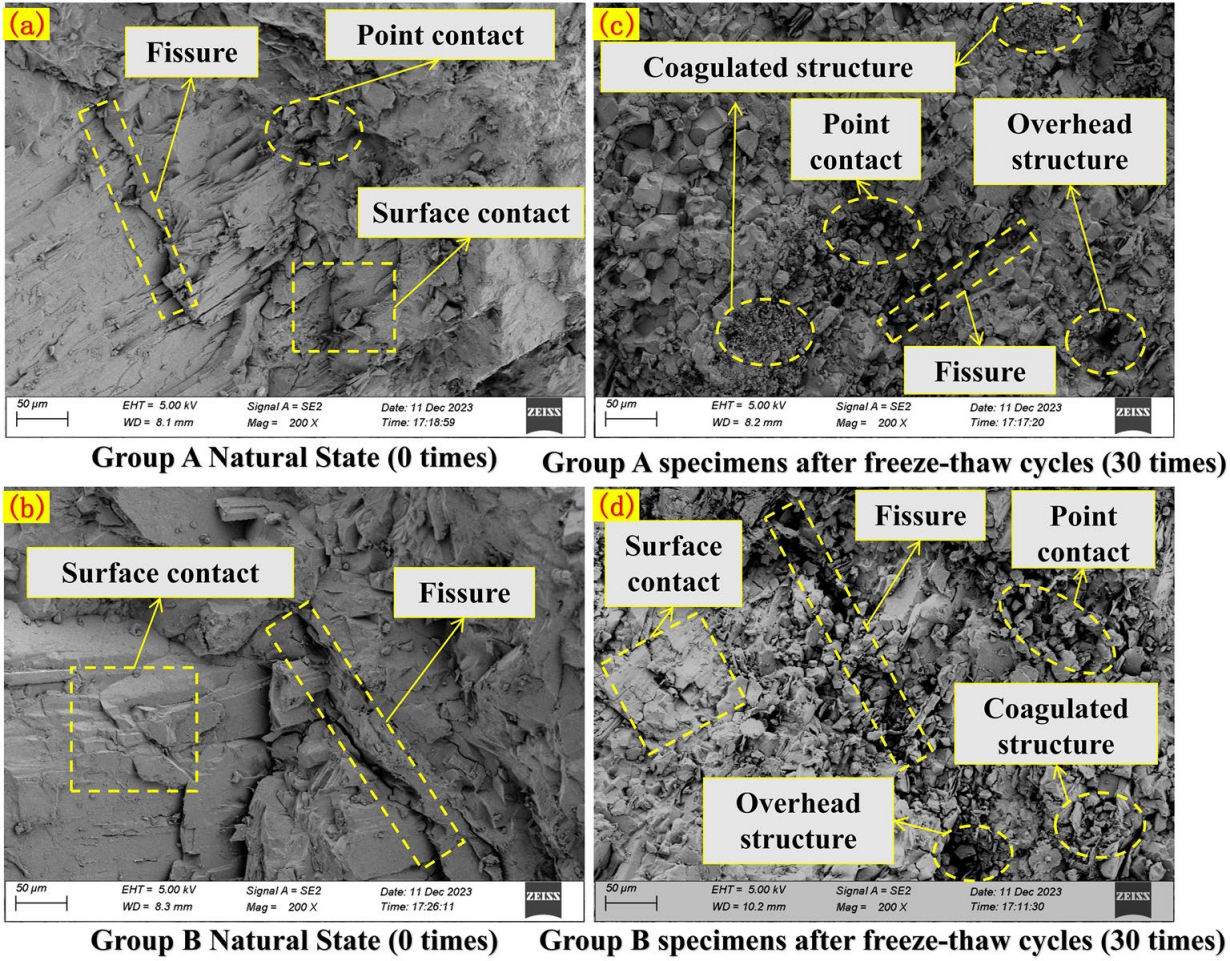
Scanning electron microscopy images of rock masses after 0-time and 30-time freeze–thaw cycles.

Scanning electron microscopy (SEM) analyses are employed on the examined specimens after freeze-thaw cycles and uniaxial compression strength tests. Specifically, SEM images at 200x magnification of samples from groups A and B, subjected to 0 and 30 freeze-thaw cycles, respectively, are scrutinized (Fig. 13).

After freeze-thaw cycles, large rock mass particles broke into smaller ones, and increased pores are evident (Fig. 13c,d). The particles aggregate into a flocculent structure, and fractures become rougher, indicating looser particle arrangements. The connection between particles changes from original surface cementation contact to point contact under freeze-thaw cycles. Comparing original rock specimens (Group A) and prefabricated surface joints (Group B), it is evident that specimens in Group B mainta in surface cementation contact after freeze-thaw cycles, while Group A specimens mostly exhibit point contacts. The destruction caused by prefabricated surface joints leads specimens to follow existing joint fractures during unidirectional loading, maintaining a surface cementation contact mode.The face cemented contact allows the rock to better resist unidirectional loading, i.e. the face cemented contact is relatively stable and requires more energy to break it when subjected to unidirectional loading, thus the rock is more likely to be damaged along existing joints and fissures when subjected to unidirectional loading.

## Discussion

**Figure 14:**
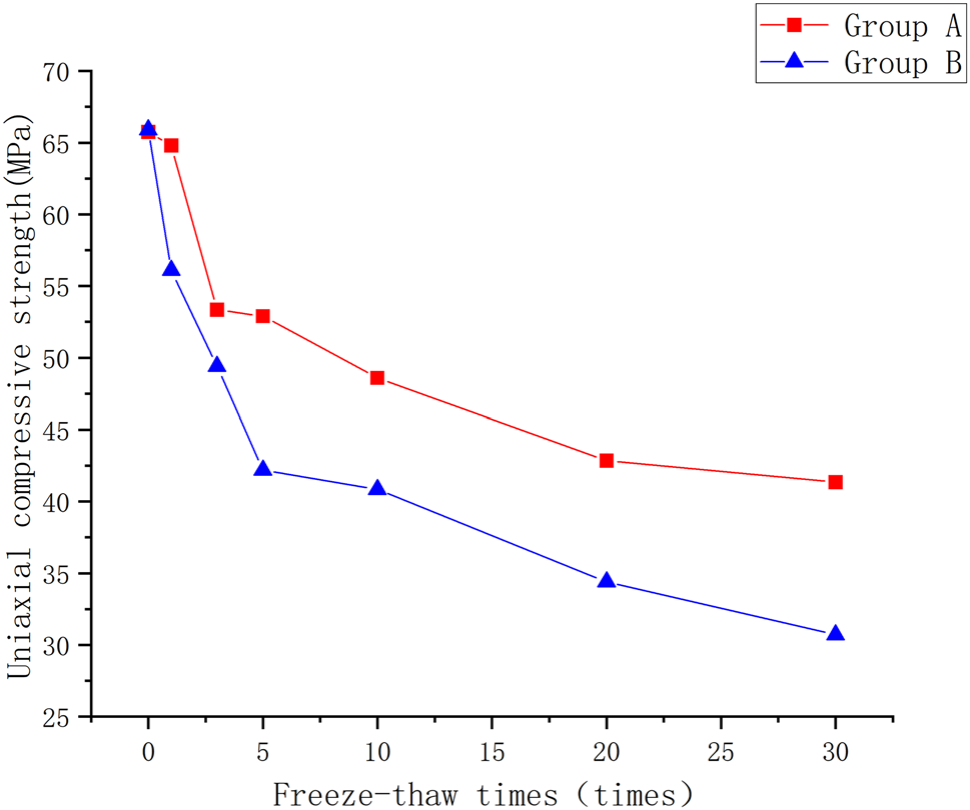
Change of uniaxial compressive strength under different numbers of freeze–thaw cycles.

This study explores the uniaxial compressive strength of both original rock masses and prefabricated jointed rock masses in relation to the number of freeze-thaw cycles to delve into the damage characteristics and mechanisms induced by freeze-thaw cycles on rock masses. Anomalies are excluded from each group's test results, and the remaining values are averaged. Figure 14 depicts the relationship between uniaxial compressive strength and the number of freeze-thaw cycles for both groups.

As the number of cycles increases, the rate of strength reduction gradually decreases, as illustrated in Fig. 14. The rock mass strength reduction rate gradually decreases with an increase in the number of cycles, as illustrated in Fig. 14. This trend is attributed to the initial freeze-thaw cycle, during which the pores in the rock mass have not yet been filled and undergo substantial expansion. As a result, the rock mass develops a stronger resistance to loading, leading to greater energy dissipation and faster reduction in strength during the early period of cycling. With an increase in the number of cycles, the addition and expansion of pore space in the rock mass result in a relatively fragile rock mass structure, making it susceptible to destruction by smaller loads during the initial stages, thus slowing down the rate of strength reduction.

The rock mass strength reduction rate gradually decreases with an increase in the number of cycles, as illustrated in Fig. 14. This trend is attributed to the initial freeze-thaw cycle, during which the pores in the rock mass have not yet been filled and undergo substantial expansion. As a result, the rock mass develops a stronger resistance to loading, leading to greater energy dissipation and faster reduction in strength during the early period of cycling. With an increase in the number of cycles, the addition and expansion of pore space in the rock mass result in a relatively fragile rock mass structure, making it susceptible to destruction by smaller loads during the initial stages, thus slowing down the rate of strength reduction.

**Figure 15:**
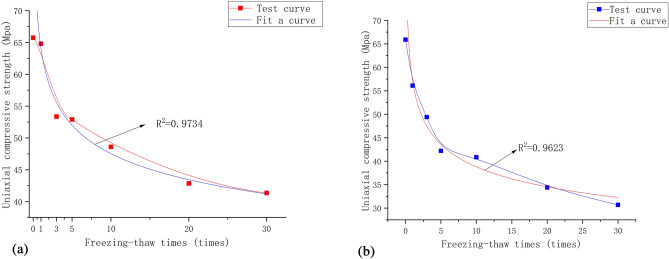
Fitting curve of uniaxial compressive strength under different number of freeze–thaw cycles. (**a**) Group A. (**b**) Group B.

The equations for the number of freezing and thawing versus uniaxial compressive strength were obtained by fitting the change curves of uniaxial compressive strength of two groups of rock samples under different freezing and thawing cycles (Fig. 15).

3 $$ {\text{Group A}}:\;\sigma = {64}{\text{.09(1}} + {\text{n)}}^{ - 0.13} $$

4 $$ {\text{Group B}}:\;\sigma = {58}{\text{.63(1}} + {\text{n)}}^{ - 0.17} $$

where: n is the number of freeze-thaw cycles.

The differences and connections between the prefabricated surface jointed rock mass and the original rock mass can be summarized. Prefabricated surface joints significantly deteriorate the rock mass, leading to a peak uniaxial compressive strength that is significantly smaller than that of the original rock mass. The damage cracks in the prefabricated surface jointed rock mass mainly result in sliding damage along the prefabricated joint surface. From a microscopic perspective, particles in the prefabricated surface jointed rock mass are mostly in direct contact with each other, and damage occurs primarily along existing cracks in the rock mass. As the number of freeze-thaw cycles increases, both groups experience a decrease in uniaxial compressive strength; however, after reaching a certain threshold, the effect of freeze-thaw on strength gradually diminishes.

**Figure 16:**
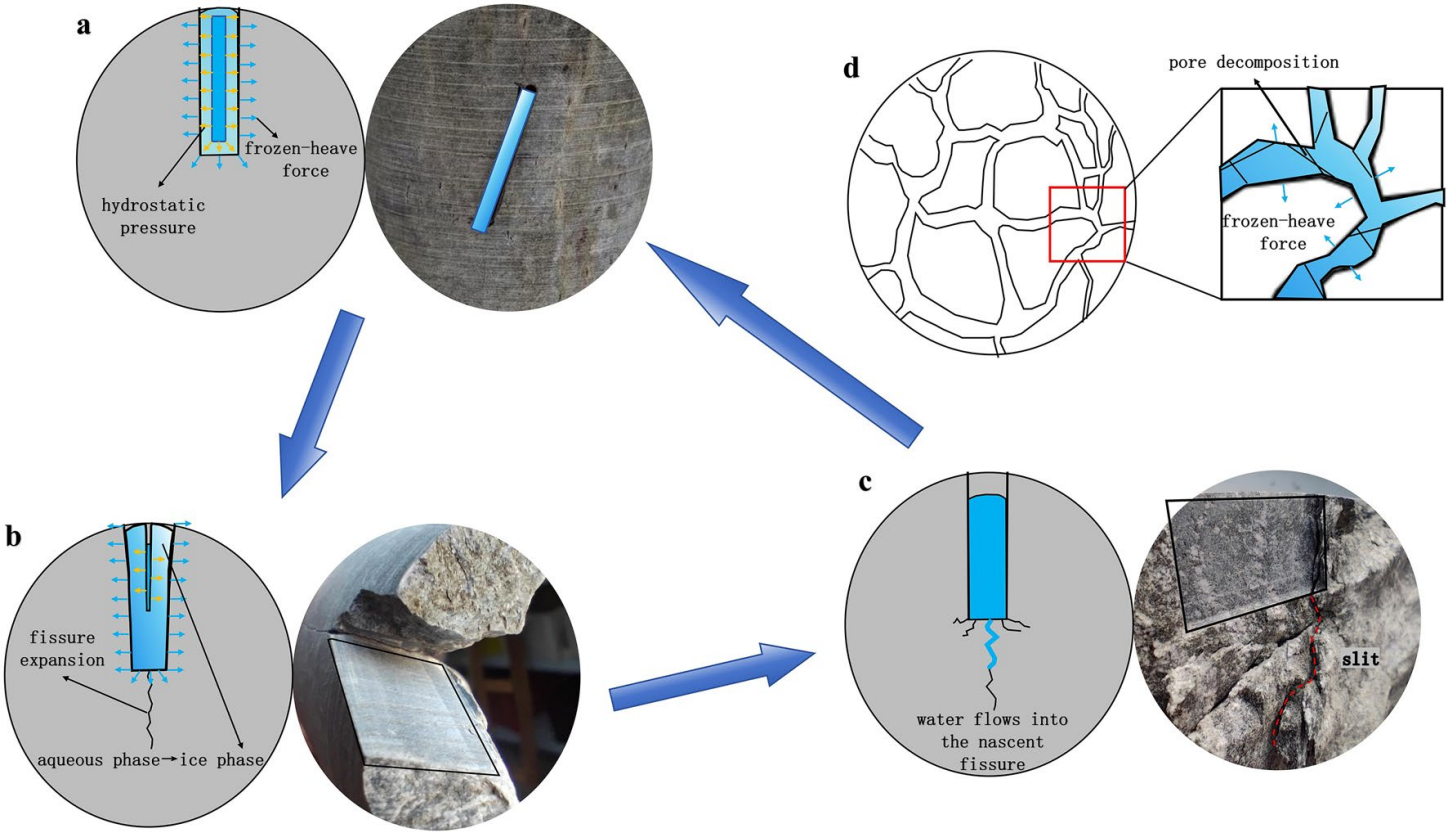
Schematic diagram of the evolution of freeze–thaw damage mechanisms in rocks.

Therefore, the mechanism of the rock mass freeze-thaw cycle is as follows: when water enters the tiny fractures and pores in the rock mass, it will freeze into ice when the temperature drops to the freezing point. When water and ice coexist, the combination of frost heaving force and hydrostatic pressure continues to act on the rock mass (Fig. 16a). As the water in the pores continues to freeze, the ice will produce about 9%^2^ volume expansion, generating pressure in the pores and further extending the fractures (Fig. 16b), which can be verified by uniaxial compression tests, where the fractures are formed at the ends of the prefabricated joints. When the temperature rises, as the ice melts, the frost heaving force decreases, and water flows into the new fractures (Fig. 16c), after being subjected to a load, the extended fractures will further expand to become through-going. As water freezes again, the process is repeated, and this repetition leads to the cracking or fragmentation of the rock mass.

As the number of freeze-thaw cycles increases, the pores in the rock mass gradually increase. When the ice in the pores is completely frozen, volume expansion will cause fractures to appear under the confinement of the pore space. Therefore, pressure will be released, leading to the breakdown of larger pores and thus reducing the effect of frost heaving forces on the rock mass (Fig. 16d). Therefore, macroscopically, it is observed that with the increase in the number of freeze-thaw cycles, the volumetric expansion pressure is gradually released until the ice expansion force becomes less than the pressure for further expansion of pores This will lead to a trend of gradually decreasing influence of the freeze-thaw action on the strength of the rock mass after the number of cycles reaches a certain range.

**Figure 17:**
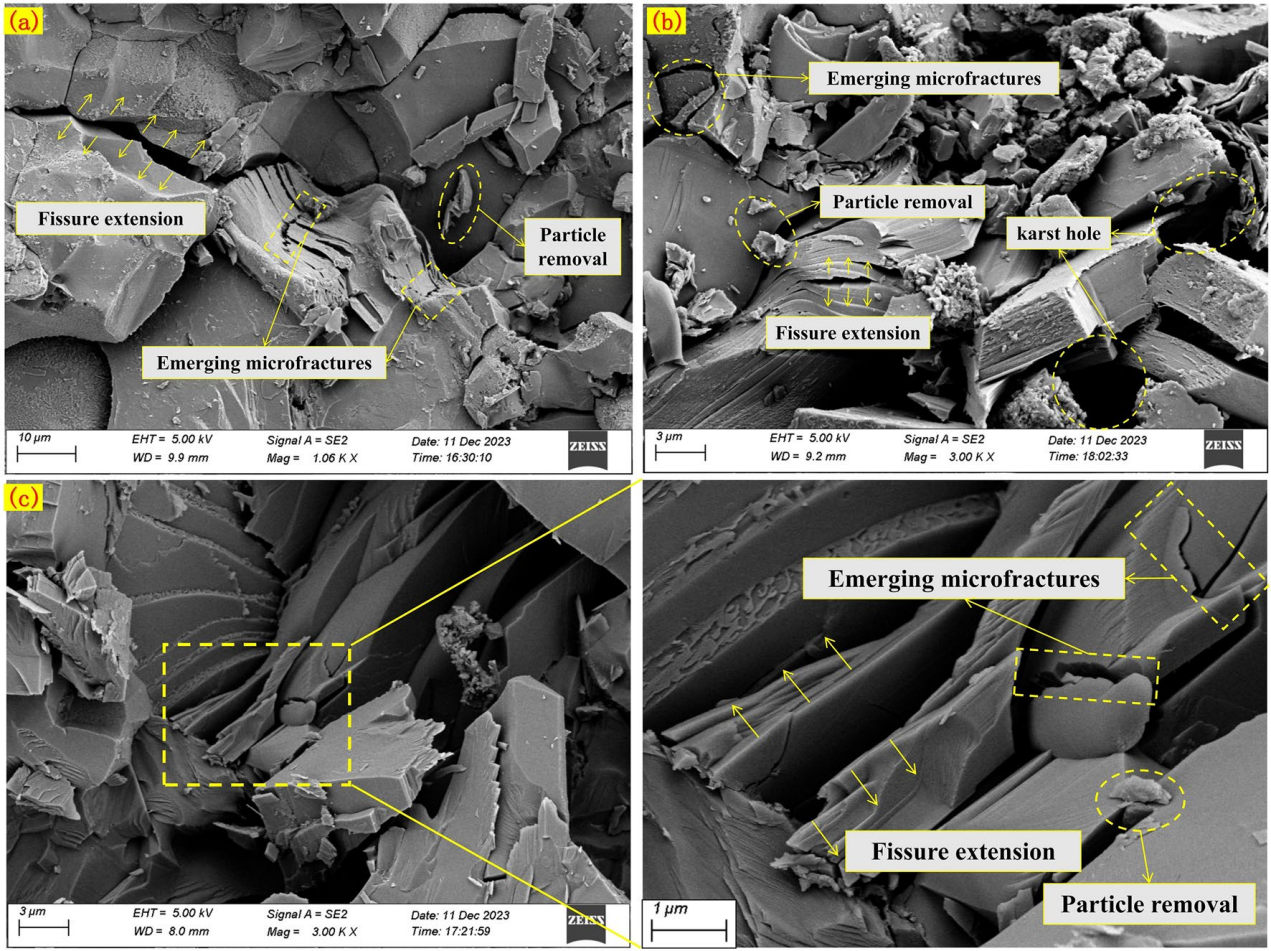
Microstructure of rock specimens after 30 cycles of freeze–thaw action.

By observing the samples after 30 freeze-thaw cycles under scanning electron microscopy, samples are taken at the edge of prefabricated surface joints for testing, and typical images are selected for analysis (Fig. 17). Due to the influence of frost heaving force, as depicted in Fig. 17a, the stacking of rock mass particles becomes loose with expanded fractures. Particle spalling occurs, and new micro-fractures appear between particles. With the increase in magnification, a small number of dissolution pores appear on the rock mass surface (Fig. 17b). In the 10,000× image (Fig. 17c), the dissolution and spalling of rock mass particles are obvious, and the phenomenon of fracture expansion is more significant. This is because the volumetric expansion of the water-ice phase change within the crack, and the repeated action of frost heaving force on the micro-cracks cause the fractures to expand, causing deeper frost damage to the more microscopic rock mass particles.

## Conclusions

This paper conducts freeze-thaw cycle tests, uniaxial compression tests, and scanning electron microscopy examinations on prefabricated surface jointed tuff specimens. The study involves observing macro-micro features before and after freezing and thawing, coupled with mechanical control tests on prefabricated joints and original rock specimens. It focuses on understanding the macro-micro features of fractured rocks post-freezing and thawing and the characteristics of fracture extension damage. The research also delves into the damage mechanisms of rocks due to freeze-thaw cycles, yielding the following key conclusions:

1. Both the mass and wave velocity of the rock samples exhibit a consistent decline with the increasing freeze–thaw cycles. The apparent characteristics of joints and fractures in the rock samples increase. Stress–strain curves for both specimen groups display extended consolidation stages.
2. The prefabricated jointed specimens are significantly more affected by freeze–thaw action both in fracture numbers and mechanical property, with a substantial decrease in uniaxial compressive strength occurring at the 5th freeze–thaw cycle.
3. Macroscopic observations reveal a gradual increase in the number of fractures with the cycling. Original rock specimens display multiple sets of wing fractures, while prefabricated surface jointed specimens show anti-wing fractures.
4. A significant deterioration effect caused by joints on the rock mass is observed. The uniaxial compressive strength of prefabricated jointed specimens is notably smaller than that of the original rock specimens.
5. After the freeze–thaw cycle, the rock mass's large particles fragment into smaller particles, forming a flocculation structure with aggregated pore particles. The particle connections shift from initial surface cementation contact to point contact under the influence of freeze–thaw cycles.

## Data Availability

The datasets used and/or analysed during the current study available from the corresponding author on reasonable request.
