# Author Correction: Deterioration mechanisms of tuff with surface fractures under freeze–thaw cycles

**DOI:** 10.1038/s41598-024-73024-4

**Published:** 2024-09-18

**Authors:** Runsen Lai, Zizhao Zhang, Jianhua Zhu, Zhiguo Xu, Xiancheng Wei, Xinyu Liu, Binghui Xiong

**Affiliations:** 1 College of Geology and Mining Engineering, Xinjiang University, Urumqi, 830017 China; 2 Research Base of Xinjiang University, National Key Laboratory of Intelligent Construction and Healthy Operation and Maintenance of Deep-Earth Engineering, Xinjiang University, Urumqi, 830017 Xinjiang China; 3 Xinjiang Geological Environment Monitoring Institute, Urumqi, 830099 Xinjiang China; 4 Xinjiang Uygur Autonomous Region Geological and Mineral Exploration and Development Bureau, Fourth Geological Brigade, Altay, 836500 Xinjiang China

Correction to: *Scientific Reports*10.1038/s41598-024-62886-3, published online 11 June 2024

The original version of this Article contained an error in the spelling of the author Runsen Lai which was incorrectly given as Rensen Lai.

The original Article has been corrected.

